# Positive beliefs about mania held by individuals with bipolar disorder: A systematic review

**DOI:** 10.1111/papt.70087

**Published:** 2026-06-18

**Authors:** Monika Luksaite, Tess Maguire, Jennifer Tarabay, Thomas Richardson

**Affiliations:** ^1^ School of Psychology, University of Southampton Southampton, Hampshire UK

**Keywords:** aspects, attitudes, beliefs, bipolar, bipolar disorder, hypomania, mania

## Abstract

**Purpose:**

To conduct a systematic review of the literature regarding positive beliefs about (hypo)mania held by individuals with bipolar disorder.

**Method:**

A systematic search of five electronic databases (PsychINFO, PsychARTICLES, MEDLINE, CINAHL and Web of Science) was carried out, using predefined search terms related to bipolar disorder, (hypo)mania and positive beliefs. Studies meeting inclusion criteria were appraised using the mixed‐methods appraisal tool. Findings were synthesised using thematic synthesis methodology due to the predominantly qualitative designs of the studies.

**Results:**

Five analytical themes were identified: (1) enjoyable feelings of (hypo)mania, (2) vitality of (hypo)mania, (3) enhanced relationship with self and others, (4) advanced ability and achievement and (5) creativity.

**Conclusions:**

This is the first review of perceived positive beliefs about (hypo)mania held by people with bipolar disorder. The findings highlight the existence of positive beliefs and provide valuable insights into the nature of such perceptions. Further research is needed to explore positive beliefs about (hypo)mania held not only by the people with bipolar disorder themselves but also by their wider network, such as family members, employers, clinicians and wider society. A better understanding of the impact and clinical implications of such beliefs is needed.

## INTRODUCTION

‘Mania is intoxicating enough to be dangerous,

original enough to be valuable.

Narrow walking indeed (Jamison, 2017)’.

Bipolar disorder (BD) is a severe and enduring mental health condition. Approximately 46 million people worldwide have a diagnosis of BD (World Health Organization [WHO], 2019). It typically involves periods of mood swings characterised by severe depression and mania or hypomania (WHO, 2019). Persistent mood swings often result in significant distress, functional and cognitive impairment, diminished quality of life (Grande et al., 2016), higher incidences of suicide (Miller & Black, 2020), and one of the biggest burdens in terms of the number of healthy years lost (Hong & Zeng, 2022).

The symptoms of mania include elevated mood, irritability, racing thoughts, exaggerated beliefs in one's abilities, increased goal‐directed activity and reduced need for sleep (WHO, 2019). This state lasts from at least four consecutive days (hypomania) to at least 1 week (mania). Despite a relatively short duration of mania, the consequences can be far‐reaching to individuals, family members and wider society. Mania is associated with risky behaviours, substance use, gambling, excessive spending (Fletcher, Parker, & Manicavasagar, 2013; Fletcher, Parker, Paterson, & Synnott, 2013), relationship problems and increased rates of suicides (Miller & Black, 2020).

Impulsive behaviours, which are common symptoms of mania, can lead to poor financial decision‐making. The range of such activities is broad; from buying a few pairs of shoes to purchasing properties and risking bankruptcy (Fletcher, Parker, & Manicavasagar, 2013; Fletcher, Parker, Paterson, & Synnott, 2013). Financially disruptive events often result in frequent financial crises and lower financial well‐being (Nau et al., 2023). Furthermore, mania is associated with relational risks; from seemingly harmless behaviours, such as flirting, to potentially regrettable, unconstrained sexual activities. Once again, the consequences are damaging for all involved: infidelity, unwanted pregnancies and potential breakdowns of the relationships. Consequently, divorce rates are higher in people with BD and so is reported loneliness (Fletcher, Parker, & Manicavasagar, 2013; Fletcher, Parker, Paterson, & Synnott, 2013).

However, despite the significant distress, the literature also indicates the existence of perceived positive aspects of BD, which are largely related to (hypo)mania (Power, 2005). The notion of positive aspects of (hypo)mania is not new; as early as the 1980s, Jamison et al. noted patients' assessment of mania as a positive contribution that improved lives in one or more significant ways. Other studies have highlighted additional perceived benefits, including enhanced functioning, greater achievement and increased creativity (Jones et al., 2006). Jamison's (1989) study of British writers and artists revealed similarities and overlaps between hypomanic and creative states. More recent research by Jones et al. (2006) found that manic symptoms correlate with a measure of inspiration. Richardson (2022) adds: ‘Mania feels like you will win the lottery, you know the cure for cancer, colours feel bright and beautiful, you have the energy to not sleep for days and not feel tired, the confidence to talk to complete strangers and crack jokes’.

Given these experiences, it is unsurprising that some individuals with BD have an ambivalent relationship with mania. By an ambivalent relationship with mania, we mean thinking and feeling that mania, although causing problems, can also be beneficial or enjoyable. It is not yet clear what proportion of people with BD have an ambivalent relationship with mania; however, emerging evidence suggests this may affect around 25%–30% of the BD population (Folstad & Mansell, 2019).

A word of caution may be needed here—that is not to say that mania is positive, but rather to emphasise the need for a better understanding of the condition and related challenges, as positive perceptions of (hypo)mania have important theoretical and clinical implications. (Lobban et al., 2012) note that unaddressed positive beliefs about mania may reduce the effectiveness of treatment or even make it unwanted. Reflecting on clinical experiences, Richardson (2022) described a bipolar relapse prevention group in which only one person (out of 10) wanted to stop mania, illustrating the potential impact positive appraisals of (hypo)mania may have on engagement with interventions.

Mania has not been well accounted for by psychological theories either. For example, the cognitive therapy model of BD is an adaptation of the classic cognitive therapy model for unipolar depression by Beck et al. (1979). The main difference is the addition of ‘bidirectional schemas' (Newman et al., 2002). Instead of a one‐directional schema, such as ‘I am unlovable’ this model proposes the polarity of schema which changes from one extreme to the other. From ‘I am unlovable to ‘everyone loves me’. The schemas tend to be either overly negative or overly positive and are activated depending on the triggering event, context, or individual's mood. However, the mechanisms of how the schemas change from one extreme to the other and trigger mania are not clear. In addition, it seems that this approach simplifies mania to the polar opposite of depression (Russell & Moss, 2013) and falls short of considering mania‐related beliefs and meanings.

A different cognitive model by Lam et al. (1999) focuses on dysfunctional beliefs and suggests that goal‐striving and anti‐dependency attitudes are particularly characteristic of BD. Elevated mood is achieved through goal‐striving attitudes such as ‘If I try hard, I should be able to excel at anything’, combined with anti‐dependency attitudes such as ‘I do not need approval from others’. This creates a positive feedback loop in which mood is elevated alongside increasing goal‐orientated behaviours and risk‐taking.

Nusslock et al. (2025) suggest that reward hypersensitivity in individuals with BD is linked to (hypo)manic symptoms characterised by excessive approach motivation. This can manifest as increased goal‐directed activity and reduced need for sleep, particularly when progress towards goals is obstructed. Both Lam et al.'s (1999) and Nusslock et al.'s (2025) models emphasise the central role of goal‐directed activity and achievement in BD. However, while this may be important for some people with BD, positive beliefs about mania incorporate many other themes, such as improved self‐esteem, and the enjoyment of ‘highs’ that are not accounted for by these theories (Power, 2005).

The amplification of positive emotions in BD is highlighted by the Positive Emotion Persistence (PEP) model (Gruber, 2011). It notes amplification of positive emotions across various contexts, including euphoria in inappropriate situations (especially during mania). The model not only highlights the persistence of feeling intense positive emotions in the BD population but also emphasises positive emotions during mania as potentially hazardous and a possible precursor to relapse. While Gruber (2011) notes potential disadvantages of positive emotions and engagement in risky behaviours, the model does not specify the underlying beliefs that may drive these emotions and behaviours.

Beliefs about mania are perhaps better addressed by the integrative cognitive model of BD and mood swings (Mansell et al., 2007). This model emphasises the role of extreme appraisals. People with BD may appraise their mood shifts in extreme ways, such as ‘I can achieve anything’ or ‘I will fail and make a fool of myself’. They then try to regulate the emotions by exerting control; engaging in ascent behaviours which further increase levels of activation (e.g., social stimulation) or engaging in descent behaviours which decrease activation (e.g., withdrawal). For example, elevated mood (internal state) can be appraised positively‐ ‘I can achieve anything when I'm high’ Such appraisal may lead to working tirelessly to achieve the goal (ascent behaviour) which elevates mood even further (Palmier‐Claus et al., 2016). Therefore, the integrative cognitive model provides a framework for how positive beliefs about mania lead to elevated mood (Richardson et al., 2024).

Nevertheless, patient experiences in clinical services suggest that positive perceptions of mania are not sufficiently considered and are often interpreted as lack of insight or resistance to treatment (Lobban et al., 2012). Current assessments and treatments are therefore predominantly focused on the negative aspects of BD (Lobban et al., 2012). Jamison (2004) noticed that psychologists attend to those in the darkness not to those in the light. Consequently, morbid emotions, such as depression, anger and anxiety, receive more clinical and scientific attention than positive ones, such as exuberance, passion for life and joy (Jamison, 2005).

This is problematic because positive beliefs about mania may reduce patients' motivation to engage in treatment and diminish treatment effectiveness. (Lobban et al., 2012). While related literature examines broader positive cognitive styles in BD, such as positive overgeneralisation, heightened confidence and elevated ambition (Richardson & Mansell, 2025), positive beliefs specific to (hypo)mania are not well understood. Therefore, we suggest that a more thorough understanding of such beliefs could provide valuable insights into the management of the condition. While there have been several individual studies reporting positive aspects of BD, to our knowledge, there have been no published reviews that synthesise the findings of research studies exploring perceived positive beliefs some people with BD hold about (hypo)mania. This review seeks to fill this gap in the existing research.

### Aims of review

1. Systematically identify research studies exploring perceived positive beliefs people with BD hold about (hypo)mania.
2. Understand what aspects of hypo(mania) some people with BD perceive as valuable or likable.
3. Assess the quality of the research studies.
4. Synthesise the findings using a thematic synthesis approach to explore perceived positive beliefs people with BD hold about (hypo)mania.

## MATERIALS AND METHODS

### Pre‐registration and search strategy

This systematic review was registered on Prospero and followed the Preferred Reporting Items for Systematic Reviews and Meta‐Analyses (PRISMA) (Liberati et al., 2009). However, there were several deviations from the protocol registered on PROSPERO. Specifically, study quality was assessed using the MMAT tool rather than CASP. This change was made due to MMAT's greater suitability for appraising studies with diverse methodological designs. Further deviations included not using the EMBASE database and two authors screening all titles and abstracts of 819 articles. The inter‐rater reliability checks were completed by a collaborator for all articles rather than a sample of 20% of articles as initially indicated. A search strategy was devised by breaking down the key components of the question into Population, Phenomena of Interest and Context (Aromataris et al., 2015). Search terms used in previous literature were considered. The search was carried out in March 2024. To ensure the search was comprehensive and accurate, free text and subject headings (where applicable) were used to search five electronic databases (PsychINFO, PsychARTICLES, MEDLINE, CINAHL and Web of Science). Additionally, the ProQuest database was searched to reduce publication bias (Boland et al., 2017). The reference lists of relevant articles were also hand‐searched. The search syntax for each database is summarised in Table 1.

**TABLE 1:** Free text and subject headings.

	(hypo)mania/bipolar disorder	Positive beliefs
Free text (All databases)	bipolar OR ‘bipolar disorder’ OR mania OR manic OR hypomani*	‘positiv* apprais*’ OR ‘positive aspect*’ OR ‘positiv* attribut*’ OR ‘positive attitude*’ OR ‘positive belief*’ OR ‘positive characteristic*’ OR ‘positive qualit*’ OR ‘positive perception*’ OR ‘positiv* experienc*’ OR ‘detrimental effect*’ OR ‘detrimental characteristic*’ OR ‘dysfunctional belief*’ OR ‘maladaptive belief*’ OR ‘creativ*’
PsychINFO subject headings	DE ‘Bipolar Disorder’ OR DE ‘Bipolar I Disorder’ OR DE ‘Bipolar II Disorder’ OR DE ‘Cyclothymic Disorder’ OR DE ‘Mania’	DE ‘Attitudes’ OR DE ‘Attribution’ OR DE ‘Irrational Beliefs’ OR ‘Positive Psychology’ OR ‘Positive Emotions’ OR ‘Creativity’
PsychARTICLES subject headings	DE ‘Bipolar Disorder’ OR DE ‘Bipolar I Disorder’ OR DE ‘Bipolar II Disorder’ OR DE ‘Cyclothymic Disorder’ OR DE ‘Mania’	DE ‘Attitudes’ OR DE ‘Attribution’ OR DE ‘Irrational Beliefs’ OR ‘Positive Psychology’ OR ‘Positive Emotions’ OR ‘Creativity’
MEDLINE subject headings	MM ‘Bipolar Disorder’ OR MM ‘Mania’	No relevant terms are available
CINAHL subject headings	MM ‘Bipolar Disorder’ OR MM ‘Mania’	No relevant terms are available
Web of Science subject headings	No relevant terms are available	No relevant terms are available

To ensure a comprehensive summary of the evidence and to reduce publication bias, the search was not limited by publication date, publication status, or language, and no methodological filters were used in order not to limit the results to specific study designs (Boland et al., 2017).

Inclusion and exclusion criteria are outlined in Table 2.

**TABLE 2:** Inclusion and exclusion criteria.

**Inclusion criteria**
Qualitative, mixed‐method and quantitative studies identifying perceived positive aspects of (hypo)mania held by people with bipolar disorder.
The search encompassed all publication dates without restriction.
Adult participants (aged 18 years old and above) with a diagnosis of BD.
Published or unpublished studies in any language with abstract available in English.
**Exclusion criteria**
Book chapters, commentaries and supplements.
Studies where the emphasis on perceived positive beliefs about hypo(mania) is deemed insufficient.
Studies exploring perceived positive aspects of (hypo)mania held by other people, such as carers, family members, employers and clinical professionals.
Children and adolescents (under the age of 18).
Participants who do not report perceived positive aspects of mania, hypomania and/or elevated mood.

### Study selection

Upon completion of the searches, Rayyan reference management software (Ouzzani et al., 2016) was used to collate the results. The search generated a total of 1177 articles: 819 after the removal of duplicates. The titles and abstracts of all 819 articles were screened by the first author and by the second reviewer (4th author) with good agreement 86.7% (Fleiss et al., 2013). Following a team discussion, 44 articles were selected for full‐text screening. Full‐text screening of relevant articles and additional studies identified through hand searching resulted in 15 papers which described nine qualitative, four mixed‐methods and two quantitative studies. The process of identification and inclusion of the studies is outlined in Figure 1, using the ‘Preferred reporting items for systematic reviews and meta‐analyses’ (PRISMA) (Page et al., 2021). Figure 1 outlines the process of selection and inclusion of relevant studies in the review.

**FIGURE 1:**
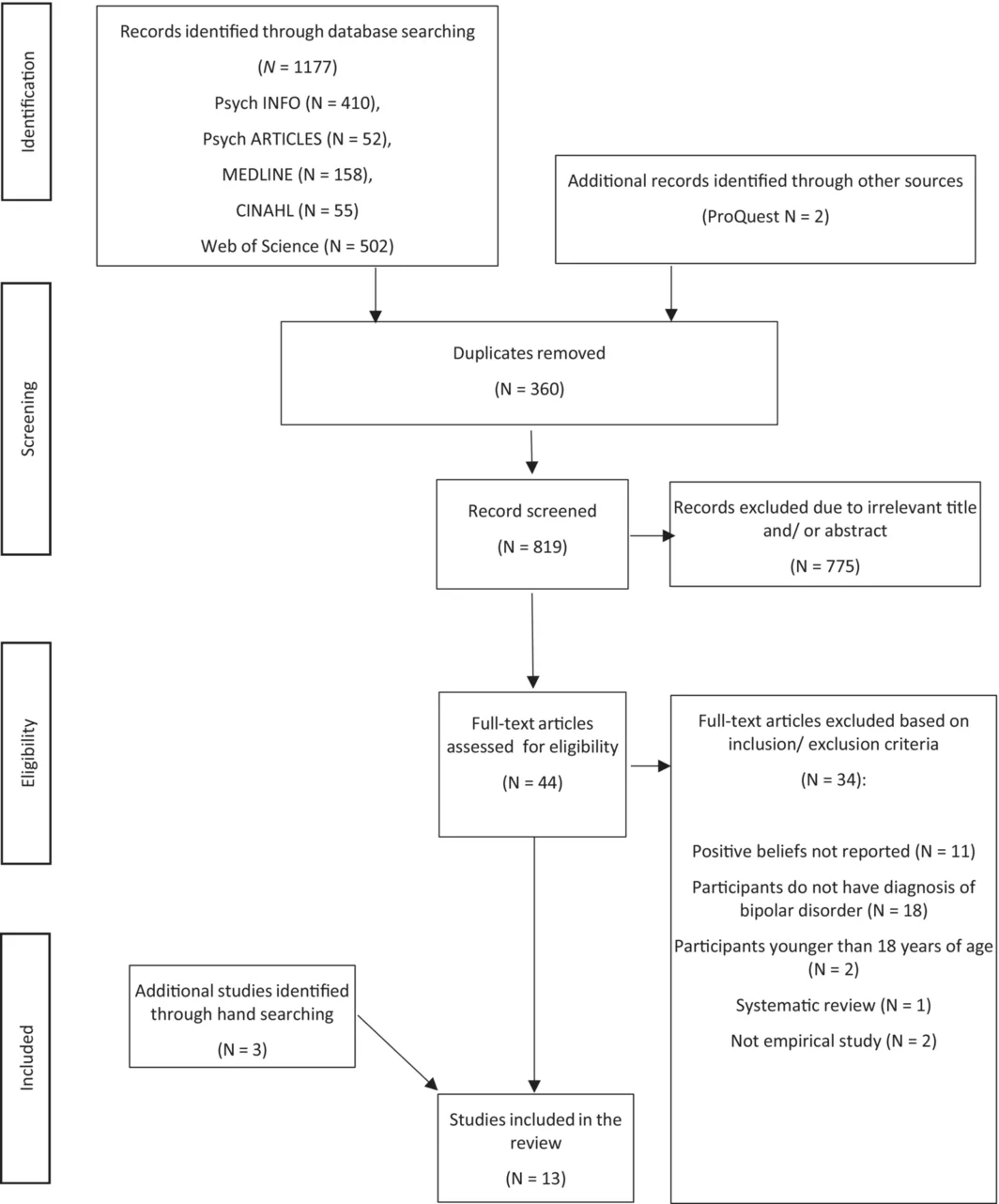
PRISMA flow diagram.

### Description of studies

The 13 eligible studies were conducted in or had corresponding authors from various countries: England (5), USA (3), Australia (3), South Africa (2), Denmark (1) and China (1). In total, there were 610 participants with BD; 385 were female and 225 were male. The age range for participants with BD was 19–64 years old. Most studies (12) were published after 2010. 61% of the studies employed a qualitative design (8). For a summary of the study characteristics, see Table 3.

**TABLE 3:** Study characteristics.

Authors	Title	Aims	Participants	Data collection	Data analysis	Reported findings
Year			Gender			
Country			Age range			
**Qualitative**						
1. Krogh et al. (2023) Denmark	Bipolar disorder and sexuality: a preliminary qualitative pilot study	To explore sexuality in connection to mood swings in five participants with BD	*n* = 5 Female = 3 Male = 2 Age = 25–46	Semi‐structured interviews	Thematic content analysis	Five themes were identified: sexual drive and impulses; sexual behaviour; thoughts and feelings about sexuality; intimate relationships; and the connection between sexuality and identity
2. Lobban et al. (2012) England	Bipolar disorder is a two‐edged sword: A qualitative study to understand the positive edge	To understand how participants with a diagnosis of BD made sense of what they took to be positive about their bipolar experiences	*n* = 10 Female = 4 Male = 6 Age = 24–57	Qualitative interviews	Interpretative phenomenological analysis	Three themes were identified: direct positive effects of bipolar on everyday experiences; lucky to be bipolar; and the relationship between self and bipolar experiences
3. Morris (2020) South Africa	Experience of persons with bipolar disorders in the context of work	To explore and make sense of the unique experiences of individuals with BD, within the South African work context	*n* = 7 Female = 4 Male = 3 Age = 23–57	Semi‐structured interviews	Thematic analysis	Four themes (and 10 subthemes) were identified: performance drivers and outcomes (sense of self, motivation, social interaction and productivity); misunderstanding and stigma (lack of understanding, unrealistic optimism and unrealistic pessimism); appropriate management (role of work, role of organisations and role of self)
4. Pagani (2018) USA	Bipolar disorder and the hypomanic experience: Creative advantages, identity, transmutation of suffering and clinical considerations among visual artists, writers and musicians	To examine the experience of creative artists with bipolar disorder, with a focus on hypomania	*n* = 7 Female = 6 Male = 1 Age = 25–65	Semi‐structured interviews	Interpretative phenomenological analysis	Five themes were identified: hypomania is correlated with multiple creative advantages; bipolar disorder intersects with creative identity on a spectrum between an integral aspect of self to a devastating medical condition; enhanced creative expression in a hypomanic state has a transformative role in counterbalancing profound depression; a history of mania adversely impacts the ability of the artist with bipolar I disorder to access the creative advantages of the hypomanic state; and treatment is essential for highly creative individuals with bipolar disorder
5. Russell and Moss (2013) England	High and happy? Exploring the experience of positive states of mind in people who have been given a diagnosis of bipolar disorder	To understand the experience of ‘happiness’ and ‘mania’ for people with ‘bipolar disorder’. To explore how they might differentiate or associate between these experiences	*n* = 4 Female = 3 Male = 1 Age = 30–60	Interviews	Interpretative phenomenological analysis	Four themes were identified: conceptual fuzziness: what is ‘happiness’? Is it being ‘high’; rejecting the Ferris Wheel idea of popular ideas of ‘happiness’ and welcoming the enjoyment and excitement of ‘being high’; the self‐acceptance and peacefulness of ‘happiness’ in contrast to the disruption and chaos of ‘being high’; the importance of community and connection in ‘happiness’ in contrast to the disruption of social relationships when ‘feeling high’
6. Taylor et al. (2015) England	Exploring the links between the phenomenology of creativity and bipolar disorder	To explore the phenomenology of and the link between extreme mood and creative activity. To explore how people with BD appraise their experiences of mood and creativity	*n* = 7 Female = 4 Male = 3 Age = 27–45	Interviews	Interpretative phenomenological analysis	Four themes were identified: high mood leads to an expanding mind; full steam ahead; a reciprocal relationship between mood and creativity; and reframing bipolar experiences through creative activity
7. Tse et al. (2014) China	Expected Possible Selves and coping skills among young and middle‐aged adults with bipolar disorder	To explore expected possible selves and coping skills among young and middle‐aged adults with BD in Hong Kong	*n* = 14 Females = 8 Males = 6 Age = 22–65	Semi‐structured interviews	Narrative inquiry methodology	Three features of positive possible selves were identified: expectations of health, paid employment and better relationships with families Young participants were hopeful about the future; middle‐aged participants experienced a sense of failure and despair
**Mixed**						
8. Folstad and Mansell (2019) England	‘The button question’: A mixed‐methods study of whether patients want to keep or remove bipolar disorder and the reasons for their decision.	To explore whether participants would or would not want to remove their BD if this was possible.	*n* = 103 Female = 80 Males = 23 Mean age = 45.9 (SD = 13.3)	Survey	Inductive content analysis Chi‐Square analyses	75 (73%) participants would permanently remove their BD if this was possible. 49 (48%) participants would remove their BD only if the effect was reversible They reported enhanced qualities and ‘fun’ of mania
9. Johnson et al. (2016) Australia, Canada and the USA	Creativity and bipolar disorder: Igniting a dialogue	To investigate how creative individuals with BD understand symptoms and treatment in relation to their creativity, and mechanisms underpinning this link	*n* = 22 Females = 17 Males = 5 Mean age = 42.05 (SD = 12.73)	Focus groups Questionnaires	Thematic analysis *t*‐tests	Five themes were identified: the pros and cons of mania for creativity; the benefits of altered thinking; the relationship between creativity and medication; creativity as central to one's identity; and creativity's importance in stigma reduction and treatment The current sample endorsed high creative accomplishment compared with the BD normative sample, and a normative community sample with no mood disorder. The highly creative sample endorsed a similar quality of life level to the normative BD sample
10. McCraw et al. (2013) Australia	Self‐reported creativity in bipolar disorder: prevalence, types and associated outcomes in mania versus hypomania	To investigate what proportion of BD patients reported creative periods during (hypo)manic episodes and when euthymic. To determine if differences existed across the BD subtypes. To examine the advantages or disadvantages of their creative periods and analyse qualitative responses.	*n* = 219 Females = 130 Males = 89 Age = 19–63	Questionnaires Interview	Chi‐square test Analysis of qualitative	82% of participants reported being creative when (hypo)manic No significant creative phenotypes specific to BD I and BD II emerged Qualitative analyses revealed 4 themes capturing advantages of creativity: good feeling; praise from others; productive/gained results; and improved focus/clarity
11. Parker et al. (2012) Australia	The ‘magic button question’ for those with a mood disorder‐ would they wish to re‐live their condition?	To determine positive aspects of living with mood disorder. To indirectly address the question – If you could live your life again, would you press the ‘magic’ button’ to experience life without a mood disorder?	*n* = 884 (BD) *n* = 111 Female = 59.9% Female (BD) = 62.6% Male = 40.1% Male (BD) = 37.4% Age = 18–81 Age (BD) = 19–64	Questionnaires and descriptions of the advantages of living with a mood disorder	Logistic regression analysis Thematic analyses	62% of participants with BD and 22.4% with unipolar depression stated that the condition had advantages such as increased empathy, self‐awareness, creativity, productivity and self‐confidence Current mood state did not impact on whether a patient nominated advantages associated with their mood disorder (wald = 2.7, *p* = .97) Five themes were identified: empathy; self‐awareness; creativity; productivity and self‐confidence
12. Parker et al. (2017) Australia	Altered Sensory phenomena experienced in bipolar disorder	To examine whether nonpsychotic sensory changes were limited to patients with bipolar II disorder or were also experienced by those with bipolar I disorder	*n* = 66 Female = 36 Male = 30 Mean age = 35.9	Interviews	NS	82% of participants experienced changes in the primary sense (smell, taste, vision, touch and hearing) during (hypo)manic or depressive states Altered sensory phenomena may be equally common in bipolar I and II disorders, and perhaps even more common in bipolar I disorder
**Quantitative**						
13. Jamison et al. (1980) USA	Clouds and silver linings: Positive experiences associated with primary affective disorders	To examine positive experiences of affective, behavioural and perceptual changes perceived by the patients. To investigate which changes during hypomania were evaluated as the most important and enjoyable. To investigate whether there are gender differences and differences between bipolar and unipolar patients	*n* = 61 *n*(BD) = 35 Female (BD) = 20 Male (BD) = 15 Age = NS	Questionnaires	Chi‐square analysis and analyses of variance	Both men and women perceived changes from normal state to hypomania, such as alertness, sensitivity, productivity and creativity. Women perceived a significantly greater increase in productivity than did men. Men found increased social ease the most important positive attribute, while women rated increased sexual intensity, increased productivity and increased social ease equally important

Abbreviation: NS = not stated.

### Quality assessment

The Mixed‐Methods Appraisal Tool (MMAT) (Hong et al., 2018) was used to assess the quality of the identified studies. The MMAT was particularly suitable for this systematic review, as it is designed to appraise the methodological quality of mixed‐methods studies, such as qualitative research, non‐randomised studies, quantitative descriptive studies and mixed‐method studies (Hong et al., 2018). The reliability of the tool is moderate (*κ* = .45) (Hong et al., 2018), whereas the validity is stated as (*κ* = .36) (Souto et al., 2015). A validity coefficient of *κ* = .36 is interpreted as low‐to‐moderate validity, and this is considered when interpreting quality ratings. The tool comprises two screening questions for all studies and five questions specific to each methodological design of the study. The manual discourages the calculation of the overall score from the ratings of each criterion and instead suggests a more detailed presentation of the ratings. This is because a single number ‘hides’ which parts of the study are problematic. Quality assessments were completed by two raters. The discrepancies were discussed and resolved with the supervisory team. Overall, the studies were of satisfactory quality. No studies were excluded based on methodological quality.

Commonly, quantitative studies relied on cross‐sectional data which precludes causal inferences. From a social constructionist perspective, however, establishing causality is not the primary concern. More salient limitations of the included studies included variable diagnostic rigour and reliance on retrospective accounts. Furthermore, the mixed‐method studies lacked clarity about the measurements (e.g., not defining what was meant by a ‘realistic’ goal or ‘creativity’).

Tables 4, 5, 6 outline the quality assessment of the qualitative, mixed methodology and quantitative studies, respectively.

**TABLE 4:** Quality assessment of qualitative studies using MMAT.

First author	Are there clear research questions?	Do the collected data allow to address the research questions?	Is the qualitative approach appropriate to answer the research question?	Are the qualitative data collection methods adequate to address the research question?	Are the findings adequately derived from the data?	Is the interpretation of results sufficiently substantiated by data?	Is there coherence between qualitative data sources, collection, analysis and interpretation?	Comments
Krogh	Y	Y	Y	Y	Y	CT	Y	*n* = 5; does not allow for subgroup analyses (e.g., gender differences) which may be important for this topic. Self‐selected participants may be more open to talking about sexual experiences than others The conclusion that sexual changes may act as a trigger, or early warning sign is not well supported by the data
Lobban	Y	Y	Y	Y	Y	Y	Y	Identified that people have a need to talk about the positive aspects of mania
Morris	Y	Y	Y	Y	Y	Y	Y	Interesting focus on South African work context
Pagani	Y	Y	Y	CT	Y	CT	Y	Participants self‐identified with a diagnosis of bipolar disorder and with a creative profession
Parker et al. (2012)	Y	Y	Y	Y	CT	CT	Y	Poor structure Included participants with ‘soft bipolar’, defined as people who do not meet the diagnostic criteria
Parker et al. (2017)	Y	Y	Y	Y	CT	CT	CT	Poor structure It is unclear how the diagnosis of BD was attained Unclear whether perceived benefits are attributable to BD The potential influence of mood state on participant recollections (not assessed for mania, which may have influenced the results)
Russell	Y	y	y	y	y	y	y	Good participant selection criteria Ethical considerations Researcher reflexivity and non‐psychiatric stance
Taylor	Y	Y	Y	Y	Y	Y	Y	Self‐selecting participants may be prone to biases
Tse	Y	Y	Y	Y	Y	Y	Y	The participants who were mentally unstable (the meaning of which was not specified) were not invited to take part, which may have led to biases

Abbreviations: BD = bipolar disorder, CT = can't tell, Y = yes.

**TABLE 5:** Quality assessment of mixed studies using MMAT.

First author	Are there clear research questions?	Do the collected data allow to address the research questions?	Is there an adequate rationale for using a mixed‐methods design to address the research question?	Are the different components of the study effectively integrated to answer the research question?	Are the outputs of the integration of qualitative and quantitative components adequately interpreted?	Are divergences and inconsistencies between quantitative and qualitative results adequately addressed?	Do the different components of the study adhere to the quality criteria of each tradition of the methods involved?	Comments
Folstad	Y	Y	Y	Y	Y	Y	Y	Self‐reported diagnosis of BD
Johnson	Y	Y	Y	Y	Y	Y	Y	Self‐reported diagnosis of BD
McCraw	Y	Y	Y	Y	Y	Y	Y	Unclear criteria for creativity The majority of patients were depressed during the study which may have impacted the results
Tharp	Y	Y	Y	Y	Y	Y	Y	No baseline measurements were taken for the acoustic recordings of goal descriptions No self‐perceptions of goal achievability were gathered Unclear definition of what a ‘realistic’ goal is

Abbreviations: BD = bipolar disorder, Y = yes.

**TABLE 6:** Quality assessment of quantitative non‐randomised studies using MMAT.

First author	Are there clear research questions?	Do the collected data allow to address the research questions?	Are the participants representative of the target population?	Are measurements appropriate regarding both the outcome and intervention (or exposure)?	Are there complete outcome data?	Are the confounders accounted for in the design and analysis?	During the study period, is the intervention administered (or exposure occurred) as intended?	Comments
Jamison	Y	Y	Y	Y	Y	No	Y	Predefined statements of potentially positive experiences of mania may have been perceived as leading by the participants.

Abbreviation: Y = yes.

### Data extraction and data analysis

Due to a small number of quantitative articles measuring heterogeneous primary outcomes, a narrative approach (rather than a meta‐analytic summary) was deemed most appropriate.

Thematic synthesis (Thomas & Harden, 2008) was chosen to synthesise the research studies and answer the review questions. This approach was preferred due to its ability to stay ‘close’ to the primary data by providing a transparent and explicit link between the text of the primary data and the generation of new interpretative constructs and explanations. Although traditionally, thematic synthesis is often associated with the synthesis of qualitative research, it can also be used to synthesise quantitative studies, particularly where there is heterogeneity in outcome variables and measurements (Ryan et al., 2018). This approach involves three stages: line by line coding of the results section of individual studies, generation of descriptive themes and generation of analytical themes (Thomas & Harden, 2008).

### Line‐by‐line coding

The first author read and re‐read the primary articles to fully immerse themselves in the data. As per Thomas and Harden (2008) all text within the results (findings) section was entered verbatim into the NVivo14 software for qualitative data analysis (NVivo 14, 2023). Each line of the text was coded inductively, capturing the meaning and content of each sentence (Thomas & Harden, 2008). Participants' quotes and authors' interpretations within the results section were treated as primary data. Following the coding, all the text with a given code was checked for consistency of interpretation (Thomas & Harden, 2008).

### Generation of descriptive themes

The codes were then grouped into descriptive themes. Such synthesis combined the findings of each study while staying close to the original findings (Thomas & Harden, 2008).

### Generation of analytical themes

This stage of the synthesis required reviewers' judgement and insight to go ‘beyond’ the content of the primary studies (Thomas & Harden, 2008). In line with this suggestion, descriptive themes were used to answer our review question. Where appropriate, the positivity of certain aspects of mania was inferred during this process and then discussed within the team. Abstract and analytical themes emerged to answer our review question. The three stages of the synthesis were carried out by the first author, who kept the records of the decisions made, as well as reflected on them with all authors during regular meetings.

The first author adopted a social constructionist position which acknowledges the absence of one truth but instead aims for a joint construction of understanding (Dallos & Stedmon, 2006). It states that objective reality does not exist. The reality is constructed through the use of language within a social and cultural context (Maturana & Varela, 1987). In clinical settings, perhaps for reasons of clinical necessity, mania is often viewed through a negative lens, with perceived positive aspects of mania often interpreted as a lack of insight or resistance to treatment (Lobban et al., 2012). Therefore, a social constructionist position helpfully offers the joint construction and understanding of multiple truths and the belief that knowledge is not predetermined but instead socially constructed (Slater, 2017).

## RESULTS

### Thematic synthesis

The findings from the 13 primary studies were synthesised into five analytical themes that answered the review question and increased knowledge of aspects of (hypo)mania perceived as positive by people with BD.

While all 13 studies contributed to the synthesis, some papers featured more prominently within specific themes due to greater conceptual richness, more detailed participant accounts or closer alignment with the review question. Studies that were referenced less frequently nonetheless contributed to the development or refinement of analytical themes.

A summary of the analytical themes and participants' and authors' quotes is presented in Table 7.

**TABLE 7:** Themes and quotes from participants and authors across studies.

Analytic themes	Papers evidencing theme	Quotes from papers
Enjoyable feelings of (hypo)mania	2, 3, 5, 6, 7, 9, 10, 11	10. Some young participants revealed that they actually enjoyed their high moods; thus, they were ambivalent about managing the manic phase
Enjoying the highs	5, 7, 8, 10, 11	8. Claire also talked about the enjoyment of ‘mania’, describing it as a state of ‘happiness’ and commenting that, ‘it is um. … good fun
Sensory amplification	2, 5, 8, 11, 12, 13	8. Karen described positively the heightened sensory experience she has when she feels ‘manic’, saying ‘everything just looked like a Van Gough painting
Vitality of mania	3, 6, 9, 10, 12	12. I have often been so excited about an involving project that I don't care about eating or sleeping
Energy	1, 5, 6, 8, 9, 11	1. Is all about just the phenomenal amount of energy
Enthusiasm	3, 6, 10, 12	12. I have often been so excited about an involving project that I don't care about eating or sleeping
Enhanced relationship with self and others	1, 2, 6, 7, 8, 10, 11, 12, 13	7. Feel connected to everything and everyone. Feel like you can have deeper conversations and more intense connections
Confidence and self‐worth	1, 2, 3, 6, 8, 10, 11, 12	12. Always felt positive and good about myself during these times and confident that this would achieve my end
Enhanced ability to understand others	2, 8, 11, 13	11. Belief that their emotional insight was greater because of bipolar disorder, resulting in an enhanced understanding of other people going through difficulties
Improved ability to interact with others	2, 3, 5, 6, 8, 11, 13, 14	14. Social ease and outgoingness‐the great majority of men and women stated that the attribute in question had either somewhat or very much increased during hypomania
Advanced ability and achievement	2, 6, 8, 10, 11, 12	6. High mood related to productivity, energy, creativity, increased confidence (contributing to social life) and belief that performance was greater in work/study situations
Ease and clarity of thought	3, 6, 8, 10, 11, 13	3. Can just come up with a solution like just like that, like a moral dilemma or an academic task or whatever and most of the time if I just stick with it, it tends to be just right
Productivity	3, 6, 8, 10, 11, 13	6. In a manic mood, uh, episode, you can do so much more
Creativity	1, 2, 3, 6, 8, 9, 10, 13	9. Participants asserted their view that to be truly creative, ideas must be original and in order to achieve this, positive affect was required to contribute towards being ever so slightly out of the zone that other people are 1. Many considered creativity to be a means of validating one's life and adding purpose to one's experience with BD

*Note*: 1 = Krogh et al. (2023); 2 = Lobban et al. (2012); 3 = Morris (2020); 4 = Pagani (2018); 5 = Russell and Moss (2013); 6 = Taylor et al. (2015); 7 = Tse et al. (2014); 8 = Folstad and Mansell (2019); 9 = Johnson et al. (2016); 10 = McCraw et al. (2013); 11 = Parker et al. (2012); 12 = Parker et al. (2017); 13 = Jamison et al. (1980).

### Enjoyable feelings of mania

The first theme ‘Enjoyable feelings of mania’ consisted of two main elements: enjoying the highs and sensory amplification.

#### Enjoying the highs

Four papers indicated that the participants enjoyed the feeling of their high moods. ‘I see it as something I really love, that gives me great sort of MDMA‐type feelings’ (Taylor et al., 2015, p. 661). They described the experience as pleasant, fantastic and gratifying (McCraw et al., 2013; Parker et al., 2012; Russell & Moss, 2013; Tse et al., 2014).

#### Sensory amplification

Six out of 15 papers identified enjoyable increased intensity of sensual experiences (Folstad & Mansell, 2019; Jamison et al., 1980; Lobban et al., 2012; Parker et al., 2012, 2017; Russell & Moss, 2013). These were as follows: enhanced visual experiences, hearing, touch, smell, taste and higher levels of sexual satisfaction. Lobban et al. (2012) reported that even when amplification was psychotic and recognised as such, it was still highly valued.

These studies describe mania as a positive experience, as well as reflect the formation of positive beliefs about manic states. Participants did not merely report that elevated moods only felt pleasant or enjoyable in the moment; they actively appraised these experiences as desirable and meaningful. For example, describing mania as something they ‘really love’ (Taylor et al., 2015), something that gives them a more intense experience of the surrounding world, suggests a broader evaluative stance rather than a short‐lived emotional response. Such beliefs extend beyond immediate emotions, shaping how individuals interpret their mood.

### Vitality of mania

#### Energy

Increased levels of energy were valued as positive by the participants in some studies (Johnson et al., 2016; Parker et al., 2012; Folstad & Mansell, 2019; Russell & Moss, 2013; Taylor et al., 2015). ‘It was just the sort of, burst of energy and everything and I just wanted to dance all the time’ (Russell & Moss, 2013, p. 438).

#### Enthusiasm

A linked but distinct aspect of heightened enthusiasm was also reported (Johnson et al., 2016; McCraw et al., 2013; Morris, 2020; Taylor et al., 2015). ‘Participants reported that, during a manic or hypomanic episode, they tend to experience a significant boost in work‐related motivation’ (Morris, 2020, p. 71).

Energy and enthusiasm described in these studies primarily reflect positive experiences of mania. However, the fact that these states were valued as positive by participants suggests the formation of positive beliefs about (hypo)mania, particularly when associated with increased work‐related motivation.

### Enhanced relationship with self and others

#### Confidence and self‐worth

Participants reported increased confidence and self‐worth (Folstad & Mansell, 2019; Krogh et al., 2023; Lobban et al., 2012; Folstad & Mansell, 2019; Morris, 2020; Parker et al., 2012; Taylor et al., 2015). Some participants described feeling more confident due to an improved ability to disregard negative feedback during sexual encounters (Krogh et al., 2023; Taylor et al., 2015), while others were more inclined to notice positive feedback received (McCraw et al., 2013; Parker et al., 2012). ‘She observed that during periods of high mood, negative feedback from others is easily ignored because the confidence induced by high mood’ (Taylor et al., 2015, p. 662).

Furthermore, people reported improved self‐worth and better realisation of the value of their contributions (Krogh et al., 2023; McCraw et al., 2013; Morris, 2020; Parker et al., 2012).

#### Enhanced ability to understand others

Individuals described increased empathy and a better understanding of others because of their mood disorder (Folstad & Mansell, 2019; Jamison et al., 1980; Lobban et al., 2012; Parker et al., 2012, 2017). This led to a better ability to connect and have deeper conversations (Lobban et al., 2012; Parker et al., 2012, 2017). ‘I am able to better relate to feelings and emotions of others going through difficult times’ (Folstad & Mansell, 2019, p. 712).

#### Improved ability to interact with others

Better social interaction was a commonly reported positive aspect of (hypo)mania (Folstad & Mansell, 2019; Jamison et al., 1980; Parker et al., 2012; Lobban et al., 2012; Morris, 2020; Russell & Moss, 2013; Taylor et al., 2015). Some participants associated improved sociability with reduced inhibitions (Russell & Moss, 2013; Taylor et al., 2015). However, other individuals emphasised the fun aspect of outgoingness and social ease (Folstad & Mansell, 2019; Jamison et al., 1980; Parker et al., 2012). ‘They enjoyed the feeling of ease with others and the perception of being an enjoyable company to their friends’ (Parker et al., 2012, p. 421).

Enhanced relationships with the self and others represent another domain in which positive aspects of mania are reported. Participants described positive changes in self‐perception, as well as enhanced empathy and a greater ability to understand and relate to others, which facilitated deeper interpersonal connections (Folstad & Mansell, 2019; Lobban et al., 2012; Parker et al., 2012). Therefore, these positive aspects of elevated mood, may reinforce positive appraisals of mania, particularly when individuals perceive themselves as more socially effective, likeable, or connected to others.

### Advanced ability and achievement

Ten studies identified advanced ability and attainment as important positive aspects of (hypo)mania. ‘In some instances, participants believed that they had achieved goals that would have been impossible without the advantages they gained from their BD’ (Lobban et al., 2012, p. 207).

#### Ease and clarity of thought

Some participants highlighted improved clarity of thought (Jamison et al., 1980; McCraw et al., 2013; Parker et al., 2012; Lobban et al., 2012; Taylor et al., 2015). ‘Everything feels clearer and I can see what is wanting to be created’ (McCraw et al., 2013, p. 834).

#### Productivity

Six papers focused on superior productivity which encompassed both increased volume of work done as well as improved quality (Folstad & Mansell, 2019; Jamison et al., 1980; 2019, McCraw et al., 2013; Morris, 2020; Parker et al., 2012; Taylor et al., 2015). They reported: ‘Increased productivity where they could accomplish many things in a short period of time, but only during “highs”’ (Parker et al., 2012, p. 421).

### Creativity

Nearly half of the papers (seven) highlighted enhanced creativity as an important and valuable positive aspect which individuals attributed to their (hypo)mania (Folstad & Mansell, 2019; Jamison et al., 1980; Johnson et al., 2016; Lobban et al., 2012; McCraw et al., 2013; Parker et al., 2012; Taylor et al., 2015). ‘Everybody I know with a mood disorder is always more creative than “normal” people. I've often wondered if it's a side effect’ (Parker et al., 2012, p. 421). Participants particularly valued the ‘expansive mind’—the ability to hold multiple streams of thought in mind as well as to think in novel and unexpected ways which they found to be the essence of their creativity (Folstad & Mansell, 2019; Johnson et al., 2016; Taylor et al., 2015).

Moreover, two papers (Johnson et al., 2016; Taylor et al., 2015) identified enhanced creativity as protective, providing participants with an important emotional outlet and method of communication and also significantly improving the quality of their recovery (Johnson et al., 2016).

Participants explicitly linked achievements to the advantages provided by mania, noting that certain goals would have been unattainable without the benefits of their high moods (Lobban et al., 2012). These accounts clearly illustrate the existence of positive beliefs about the value and functionality of manic states, particularly when individuals perceive heightened mood as facilitating enhanced performance or creative accomplishment.

## DISCUSSION

This is the first systematic review of the perceived positive beliefs about (hypo)mania held by some people with BD. A comprehensive search of published and unpublished studies generated 13 papers, the majority of which were qualitative. Thematic synthesis of the papers provided an important insight into what some individuals with BD perceived as positive aspects of their disorder.

The review identified a broad range of positive beliefs. They included enjoyable feelings of mania and sensory amplification, improved self‐worth, better ability to relate to other people and increased energy, productivity, enthusiasm and creativity. The studies capturing such beliefs were conducted in several different countries, covering a wide geographical area. The exception to this was sexuality‐related insights which, in this review, were limited to the studies from Western countries, namely the USA, the UK, and Denmark (Folstad & Mansell, 2019; Jamison et al., 1980; Krogh et al., 2023). That is not to say that the phenomenon does not exist elsewhere; however, Western countries are thought to have a more liberal view of sexual experiences (Krogh et al., 2023), and it is possible that people were more open to sharing them.

Overall, the themes identified in this review fit well with existing cognitive theories of BD. Although existing cognitive models of BD largely overlook positive beliefs about mania, our findings indicate that these beliefs can be meaningfully integrated into current models by refining and extending them. For example, the themes ‘Advanced ability and achievement’, ‘Ease and clarity of thought’, ‘Productivity’ and ‘Creativity’ seem consistent with cognitive models of BD; particularly the cognitive model by Lam et al. (1999) which emphasises goal‐striving and anti‐dependency attitudes. This model explains elevated mood through increasingly goal‐driven behaviours and risk‐taking. As illustrated by previously provided quotes, participants valued high mood related productivity, clarity and ease of thought, and the ability to achieve more, and with less effort. Elevated mood led to increased achievement which in turn further elevated mood (positive feedback loop). Consistent with the cognitive model, participants were less sensitive to potentially negative feedback and less likely to be inhibited (anti‐dependency attitude) (Lam et al., 1999).

Although the cognitive model provides a clear mechanism of mood elevation, it seems to be rather simplistic (Power, 2005) and perhaps lacks depth and a more nuanced understanding of people's motivations. Our findings indicate that participants believed mania was necessary to elevate mood, enabling them to accomplish goals that would have been otherwise unattainable. This perception may, in turn, reinforce the pursuit of (hypo)manic states, as individuals seek the emotional and cognitive advantages they associate with these episodes.

We suggest that understanding such perceptions is of great importance.

Our findings also extend Gruber's (2011) understanding of positive emotions by highlighting how individuals with BD perceive (hypo)mania not only as a source of intense positive affect but also as a means to achieve personally meaningful goals. The theme ‘Enjoyable Feelings of Mania’ further illustrates that participants' experiences were not merely descriptions of elevated mood; rather, they often framed these experiences as meaningful counterweights to depressive suffering. Enjoyment was therefore not just a pleasurable experience, but it was frequently embedded within narratives of relief, vitality, and reclaimed identity.

Similarly, in our review, the theme ‘Creativity’ uncovered an important additional nuance: that people valued the pursuit of creative encounters as a way of validating their lives, a way of adding purpose to their lives and seeing themselves beyond the illness. Participants found creativity to be of protective value; they embraced creativity as an emotional outlet and appreciated the positive effect it had on their recovery (Johnson et al., 2016).

Moreover, ‘Enjoyable feelings of mania’, ‘Sensory amplification’, ‘Vitality of mania’, ‘Energy’ and ‘Enthusiasm’, seem consistent with the cognitive integrative model (Mansell et al., 2007). This model suggests that people with BD appraise their mood shifts in extreme ways and then regulate their emotions by exerting control and engaging in ascent or descent behaviours. In the case of mania, the ascent behaviours are of more relevance. Activating behaviours were evident in the participants' accounts in the current review. People shared their experiences of being more social, more outgoing, being the soul of a party and the problem‐solver at work. However, not all ascent behaviours were helpful. Consistent with the research, the ascent behaviours involved unhelpful and risky behaviours too. They ranged from regrettable and spontaneous sexual activities to poor financial decision‐making, manifesting in excessive spending and buying unnecessary things (Russell & Moss, 2013).

Because the insight diminishes in (hypo)mania and the consequences accrue later, it may not be clear to the patient that the costs of mania often outweigh the benefits. Therefore, it is important that clinicians support patients in recognising and working on positive beliefs about mania.

While a strong human desire to experience happiness is understandable, and often seen as an indicator of psychological health, BD and particularly (hypo)mania provide a unique clinical opportunity to consider how persistence on positive emotions could be unhelpful and lead to devastating consequences (Gruber, 2011).

Despite its importance, few studies have explored the clinical significance of positive beliefs of (hypo)mania in BD. This review, therefore, adds new insights, gives a voice to people with BD, and listens to their experiences of mania. It highlights several common themes across the studies, spells out specific aspects of mania that individuals with BD identify as positive. As previously stated, the aim of this review is not to suggest that hypo(mania) is fun, enjoyable and ability‐enhancing quite the opposite, as the consequences of BD can be devastating. The reason it is important to gain a good understanding of what people with BD perceive as positive about their disorder is that this may carry important clinical implications (discussed in the paragraph below). Therefore this review highlights the need to broaden our attention both academically and clinically and consider including positive aspects of mania in our thinking, therapeutic work and research (Lobban et al., 2012).

### Limitations and future research

This review used a systematic methodology to identify, appraise and synthesise research studies indicating the existence of positive beliefs about mania held by some people with BD. However, the findings should be interpreted within the confines of several limitations.

Overall, most of the studies were of satisfactory quality. They had clearly formulated research questions, appropriate data collection, analyses and insightful interpretations of the findings.

However, participant inclusion criteria varied across the studies. This was particularly relevant for the assessment of the diagnosis of BD. Only a minority of included studies explicitly reported the use of standardised diagnostic interviews (e.g., SCID), while others relied on self‐reported diagnoses or clinical records.

In addition, reporting of bipolar subtype was inconsistent. Where specified, samples were typically composed of individuals with both bipolar I and bipolar II disorder, with few studies reporting subtype proportions separately. This limits the extent to which findings can be interpreted in relation to differences between manic and hypomanic experiences.

While hypomania may facilitate idea generation, mania could impair execution and output quality (da Cruz et al., 2022). Current research has not sufficiently distinguished hypomania from mania to draw firm conclusions.

Furthermore, several studies recruited participants from artistically identified or highly creative populations. While such samples provide valuable insight into specific lived experiences, they may also reflect selective strengths or values that are not representative of the broader bipolar population. This limits generalisability and should be considered when interpreting findings related to creativity and achievement.

Another important limitation concerns the developmental trajectory of positive beliefs about (hypo)mania. Studies involving younger participants were excluded from the present review, which limits insight into how such beliefs may emerge, consolidate, or change across the lifespan. Only a few studies reported illness‐course variables such as number of episodes or hospitalisations. It is plausible that repeated exposure to the adverse consequences of mania may attenuate positive beliefs over time, whereas earlier illness stages may be associated with greater ambivalence. Future research should explicitly address these factors.

Overall, the studies gave little consideration to gender differences, ethnicity, culture, or special populations (e.g., pregnant people). Early accounts (Jamison et al., 1980) point out gender differences in what was regarded as most valuable when hypomanic; for example, increased social ease was most important for men, whereas women rated increased sexual intensity, productivity and social ease as equally important. Additionally, Jamison (2005) noticed gender differences in societal expectations in the context of mental health difficulties; she states that depression seems more compatible with what society expects from women: sensitive, passive and stricken, whereas manic states seem more ‘masculine’; aggressive, risk‐taking, grandiose and visionary. While such stereotypically genderised adjectives could be debatable, it is possible that gender differences would be seen in perceived positive beliefs about mania. Therefore, it would be important for future research to disentangle positive beliefs about mania taking gender and other individual differences, such as ethnicity, and culture into account.

The review aimed to be comprehensive; it identified and included a broad range of relevant studies and did not exclude studies based on their publication status or language. However, only the studies with the views of people with a diagnosis of BD were included. That is, positive beliefs about mania held by other people, such as family members, carers, clinicians and wider society were not included. Current research indicates that family members, employers and wider society sometimes hold positive beliefs about mania too (Fletcher, Parker, & Manicavasagar, 2013; Fletcher, Parker, Paterson, & Synnott, 2013; Pagani, 2018). This may reinforce the views that people with BD hold and have important clinical implications. It is therefore important to understand positive beliefs about mania across wider networks and the potential impact this may have.

Furthermore, only ProQuest was searched for unpublished literature. This may have narrowed the results and may be subject to bias.

## CONCLUSIONS

The present findings emphasise the existence of positive beliefs about mania held by some people with BD. It is our hope that this summary will help people with BD, their loved ones, and their clinicians to gain a better understanding of common experiences of mania.

It is important for clinicians to be aware of the existence of positive beliefs in order to be therapeutically more effective (Jamison et al., 1980) in helping people to recognise and challenge positive beliefs about mania. It may be worth noting that clinicians are faced with a unique challenge here, as it is more common to challenge negative cognitions.

To date, research and clinical work has been focused predominantly on the negative aspects of BD. Being attentive to people's positive experiences of mania may help to improve our understanding of ambivalence to treatment in BD. It would be logical to think that someone who sees their difficulty as beneficial would not adhere to treatment, at least until the consequences become severe. Unaddressed positive beliefs about mania can make the treatment less effective or unwanted (Lobban et al., 2012), therefore, a good understanding of positive appraisals of (hypo)mania is important and clinically relevant. This review provides valuable insight into what such beliefs may look like.

It is important for clinicians to consider valued aspects (objective or not), moving away from a predominantly negative focus and interventions that mainly consider symptoms of depression (Taylor et al., 2015).

The reviewed literature demonstrates that the positive aspects of mania encompass more than fleeting pleasurable experiences, extending to beliefs about the value and functionality of elevated mood. Clinically, this is important: such beliefs may contribute to the pursuit or maintenance of (hypo)manic states and can influence attitudes towards treatment. Recognising and addressing the valued aspects of (hypo)mania in therapeutic settings may therefore improve engagement with interventions while promoting safer mood regulation strategies.

This review extends current theoretical models of BD by incorporating the role of positive beliefs about mania. Integrating these beliefs into theoretical frameworks may support a more nuanced understanding of the cognitive and motivational processes underpinning BD.

We hope that this review will inspire researchers to investigate this subject further. It would be helpful for future research to expand on this review and investigate positive beliefs about mania across wider networks and society, and to take individual differences into account. Future studies would benefit from the consistent use of standardised diagnostic assessments, clearer reporting of bipolar subtypes, and greater differentiation between mania and hypomania. Research should also aim to recruit more representative samples and examine the developmental trajectory of positive beliefs across the lifespan, including the role of illness‐course variables such as episode frequency and severity. Addressing these limitations represents an important next step towards advancing a more comprehensive and clinically meaningful understanding of positive beliefs about (hypo)mania in BD.

## AUTHOR CONTRIBUTIONS

**Monika Luksaite:** Conceptualization; investigation; writing – original draft; methodology; formal analysis; data curation. **Tess Maguire:** Conceptualization; investigation; methodology; writing – review and editing; formal analysis; data curation; supervision. **Jennifer Tarabay:** Writing – review and editing; formal analysis; data curation; conceptualization; investigation. **Thomas Richardson:** Conceptualization; investigation; methodology; validation; writing – review and editing; supervision.

## CONFLICT OF INTEREST STATEMENT

The authors declare no conflict of interest.

## Data Availability

The data that support the findings of this study are openly available in eprints.soton.ac.uk at https://eprints.soton.ac.uk/493888/, reference number 493888.
